# Supplementary material for: Analyzing temporal and spatial forest carbon storage using Google Plus Code: a case study of Zijin Mountain National Forest Park, China
Source: Carbon Balance Manag. 2024 Apr 15;19:13. doi: 10.1186/s13021-024-00258-0 (PMC11020297; doi:10.1186/s13021-024-00258-0)
Supplement: Supplementary file 1 — Supplementary Material 1 [file 13021_2024_258_MOESM1_ESM.docx]

Analyzing temporal and spatial forest carbon storage using Google Plus Code: a case study of Zijin Mountain National Forest Park, China

Xiaorong Wen^1, 2^，Li Yang^1, 2^, Yanli Zhang^3^, Qiulai Wang^2, 4^, Jinsheng Ye^2, 4^, Matthew McBroom^3^

^1.^ Co-Innovation Center for the Sustainable Forestry in Southern China, Nanjing Forestry University, Nanjing, Jiangsu 210037, China

^2.^ College of Forestry, Nanjing Forestry University, Nanjing, Jiangsu 210037, China

^3.^ Arthur Temple College of Forestry and Agriculture, Stephen F. Austin State University, Nacogdoches, TX, 75962, USA

^4.^ Guangdong Forestry Survey and Planning Institute, Guangzhou, Guangdong 510520, China

Corresponding author: Yanli Zhang, [zhangy2@sfasu.edu](mailto:zhangy2@sfasu.edu)

Yanli Zhang’s ORCID: 0000-0002-6887-3575

Analyzing temporal and spatial forest carbon storage using Google Plus Code: a case study of Zijin Mountain National Forest Park, China

**Abstract:**

**Background** It is always a challenging job to compare forest resources as there is not a standardized spatial unit with location information. Google Plus Code, the newest alphanumeric geocoding system, uses 20 specifically selected letters and numbers to assign a unique global ID to every cell at different levels of a hierarchical grid system which is established based on latitude and longitude. It can be used as a standardized, unique global geospatial unit to segment, locate, quantitate, evaluate, and compare natural resources with area, boundary, and location information embedded.

**Results**  For this proof-of-concept case study, forest inventory data from 1987, 2002, and 2019 for the Zijin Mountain National Forest Park in Jiangsu Province, China was analyzed based on Google Plus Code grid/cell. This enabled the quantification of carbon storage at each cell allowing for the comparison of estimated carbon storage at same or different locations over time.

**Conclusions** This methodology is used to quantify the impacts of changing forest conditions and forest management activities on carbon storage with high spatial accuracy through the 32-year study period. Furthermore, this technique could be used for providing technical support and validation of carbon credit quantification and management.

**Key Words:** alphanumeric geocoding system, Google Plus Code, carbon storage, spatial distribution

**1. Background**

Sustainable forest management at the global, national, and regional scales requires timely and accurate forest inventory data that are georeferenced using a standardized spatial unit with the appropriate location information embedded. This need is particularly acute when it comes to measuring and estimating forest carbon storage for climate change mitigation strategies [1-4]. Carbon storage is commonly estimated based on forest biomass volumes or growth rates being multiplied by the appropriate carbon index [5-7]. Beyond total carbon storage estimates, the spatial distribution of the carbon stored within a forest is necessary to manage forests for optimum carbon storage [1, 6, 8]. From these spatial distribution patterns, a cartographical presentation of forest carbon storage can be useful to provide stakeholders and decision makers better tools for making the most appropriate policy decisions or economic investments. However, these cartographic analyses require widely available and accurate georeferenced spatial units.

Commonly used spatial units like street addresses are limited for defining stand conditions in forestry applications and latitude/longitude are applicable only to specific spatial points [9-11]. Techniques like the Kriging geostatistical method have been successfully used to develop forest carbon storage maps [8]. In addition, the spatial error model (SEM) has been employed to relate the distribution of forest carbon storage to both forest stand parameters and topographic characteristics [12]. Geospatial regression has also been employed as a tool for relating the spatial distribution of biomass estimates and carbon storage in forests [6]. While Kriging, SEM, and geospatial regression have been applied for cartographic representation of forest carbon storage, these previous carbon storage studies usually analyze a large area of forest all together without breaking the study area into comparable and geolocatable spatial units, thus they are not helpful for on the ground carbon storage monitoring and management.

A novel technique has recently become available for a wide variety of geospatial applications, the use of Google Plus Code as an alphanumeric geocoding hierarchical grid system. This system provides exact spatial locations and unique global identification over wide ranging areas. When comparing with traditional unregular plot or sub-compartment boundaries, Plus Code cells provides foresters a more convenient and accurate geolocating way for on the ground monitoring and management. However, the applicability of this grid system for forest carbon storage has yet to be developed and demonstrated for temporal and spatial forest dynamics. Therefore, this research project was initiated utilizing forest inventory data from 1987 through 2019 in one of China’s most iconic scenic natural areas, Zijin Mountain National Forest Park. These data were employed to determine georeferenced carbon storage utilizing Google Plus Code, with the purpose of providing researchers and decision makers with an effective method for long-term forest carbon stock management.

**Methods**

Study Area

Zijin Mountain National Forest Park is located in Nanjing, Jiangsu Province, China. The area’s latitude and longitude ranges from 32°01’57”N to 32°18’15”N and from 118°48’24”E to 118°53’04”E, respectively. The highest elevation in the park is 448.9 m and it has an area of 3,008.8 ha. Approximately 76.8% (2,311.1 ha) is forested. Due to the mid-latitude location, the region has a typical northern subtropical climate with about 2,213 hours of sunshine each year and an annual average temperature of 15.4℃. The rainy season is typically in June and July and average annual precipitation is 1,090.4 mm, with 70% occurring during spring and summer and 30% during fall and winter. Soils are mostly acid or weak acid yellow brown Dystric Cambisols and Eutric Planisols. Evergreen broadleaf species are the dominant canopy trees in the forest with some deciduous broadleaf species and bamboo. The predominant species include Masson pine (*Pinus massoniana* Lamb), Formosan gum (*Liquidambar formosana* Hance) and various oak species (*Quercus* spp.).

Forest Inventory Field Data Collection

The forest in this research is where there is a canopy within the park and forest management inventory were conducted in 1987 and 2002. These historic data were obtained from the Zijin Mountain National Forest Park administration database. From May to December in 2019, a third forest inventory was conducted. In total, there were 777 sub-compartments or stands delineated within the study area (Figure 1 and 2). Sampling or measurement plots were 666.67 m^2^ (25.82 m x 25.82 m) with approximately one plot per three ha, based on compartment size. At each measurement plot, aspect, slope, soil type, soil depth, species, age, age class, diameter at breast height (DBH), height, and canopy closure were recorded. Forest condition and tree volume were then estimated for each sub-compartment based on these measurements.


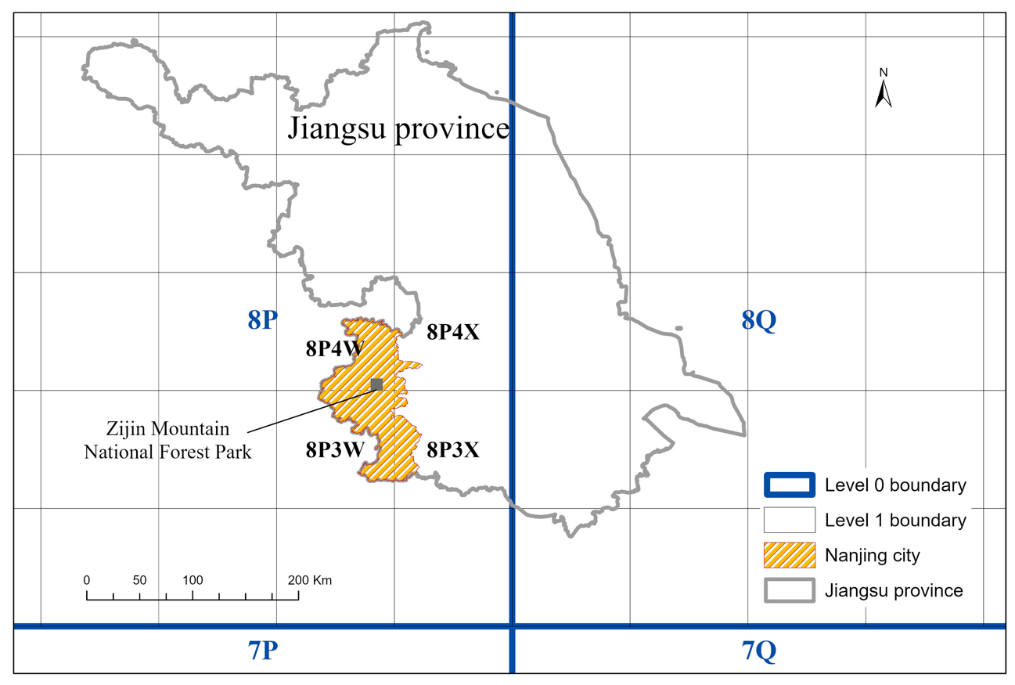


**Fig. 1** Google Plus Code level 0 (2-digit) and level 1 (4-digit) grids and location of the Zijin Mountain National Forest Park in Jiangsu Province, China


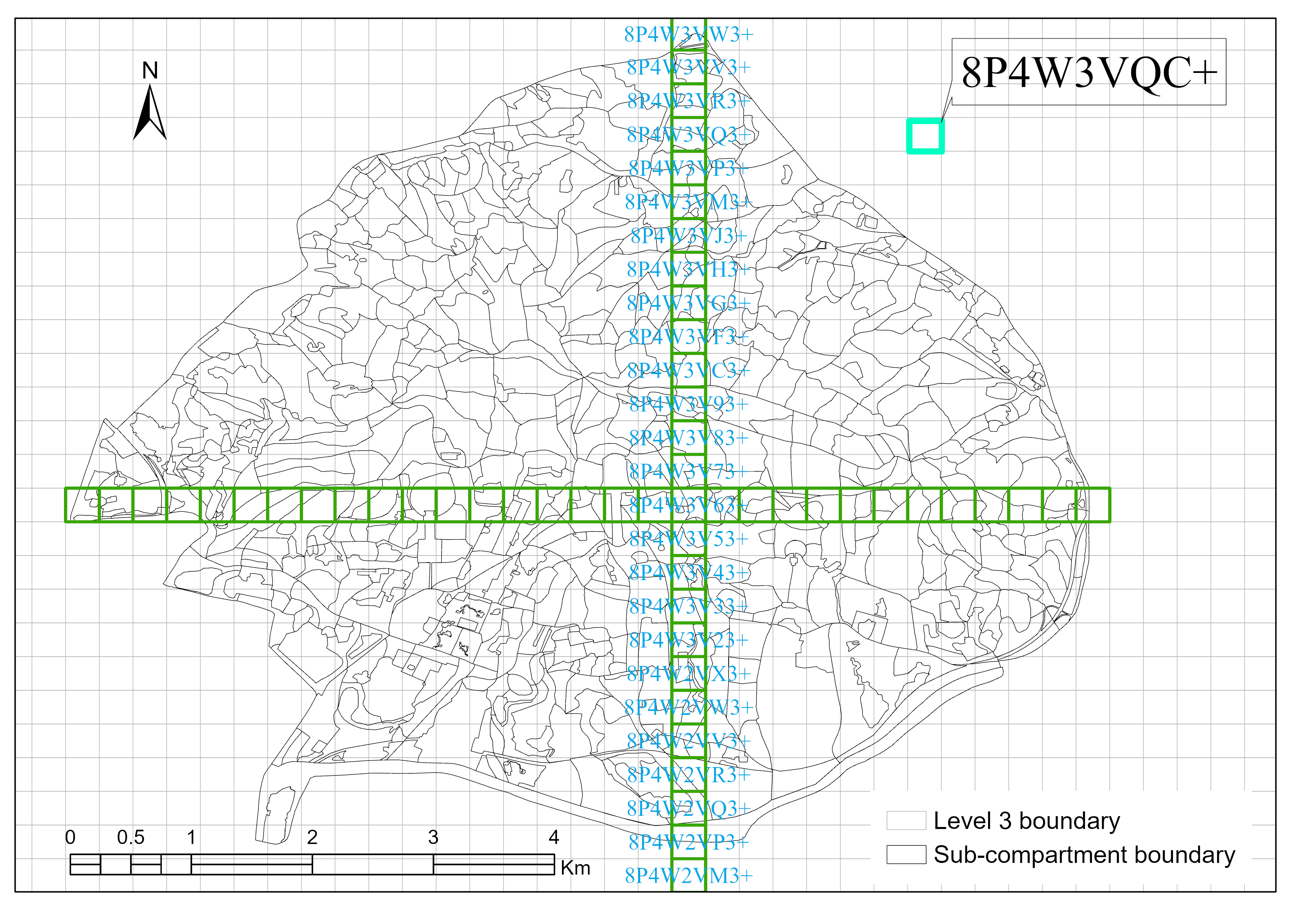


**Fig. 2** Google Plus Code level 3 grid (8-digit) and the forest sub-compartments of the Zijin Mountain National Forest Park in Jiangsu Province, China

Estimation of Forest Biomass and Forest Carbon Storage

For estimation of forest biomass, the method described by Fang et al. [4] was used with the biomass conversion continuous function with coefficients being described by:

$B=aV+b$ (1)

Where B is the forest biomass (t, metric ton), V is the forest volume (m^3^), a and b are conversion coefficients which change depending on forest type or tree species. Measurements of litter, understory, and grass biomass were not available so only above-ground biomass was estimated in this study. The a and b values are adopted from researches done by Zhang et al. [13], Zhao et al. [14] and Fang et al. [5] and are summarized in Table 1.

Table.1 Biomass conversion equations and carbon content rates for forest cover types in the Zijin Mountain National Forest Park in Jiangsu Province, China.

| Forest type | Equation | Carbon content (*q_i_*) |
| --- | --- | --- |
| Locust (*Robinia pseudoacacia*) | B=0.7564V+8.3103 | 0.480 |
| Oak (*Quercus*) | B=0.7848V+16.715 | 0.500 |
| Formosan gum (*Liquidambar formosana Hance)* | B=0.4754V+30.6034 | 0.497 |
| Masson pine (*Pinus massoniana Lamb)* | B=0.6632V+7.2656 | 0.460 |
| Mixed conifers | B=0.7442V+26.806 | 0.510 |
| Mixed broadleaves | B=0.7393V+43.21 | 0.490 |
| Mixed (Mixed Conifers and broadleaves) | B=0.4385V+52.905 | 0.498 |
| Bamboo *(Phyllostachys pubescens)* | B_b_=81.9S_b_ | 0.500 |

*B is the forest biomass (t), V is the forest volume (m^3^), B_b_ is the bamboo biomass (t), S_b_ is the total area of bamboo forest (hm^2^) [17].

Forest carbon content (*q_i_*, Table 1) was obtained from the Guide for Carbon Accounting and Monitoring in Afforestation Projects which utilized the Intergovernmental Panel on Climate Change (IPCC) reference values and bamboo forest carbon content of 0.50 [15, 16]. The formula used to calculate forest carbon storage (C) is:

$C=q_{i}\times B$ (2)

Forest carbon density is the forest carbon storage per unit area and was calculated as:

$\rho=C/S$ (3)

$\rho$is forest carbon density (tC/ha), C is carbon storage (tC) and S is the corresponding spatial area (ha).

Geospatial Distribution of Forest Carbon Storage Using Google Plus Code

There is currently not a standard geospatial unit with embedded location information that is widely utilized for forest management. In recent years, alphanumeric geocoding systems, such as What3Words, Mapcode, and Placekey, were developed to locate any point on Earth. The newly available Google Plus Code has a similar utility and is based on the Open Location Code (OLC). Although none of these geocoding methods were designed for forestry applications directly, the hierarchical grid system of Plus Code enables foresters to have comparable cells for the entire world. Google Plus Code uses latitude and longitude to start the level zero grid (20° by 20°) and each cell has a two-digit (letter and number) ID. Only 20 specifically selected letters and numbers are used to prevent from confusion that can occur between certain alphanumeric pairings, such as lower-case L and the number one. Each 20° by 20° macro-cell is then sub-divided into level one sub-grids (1° by 1°). At this level, each cell will have a unique four-digit ID (two digits from level zero and two digits for level one itself). This sub-division is further sub-divided until a level five (12 digits ID) is determined. A different structure is used for level six+ divisions (Figure 1). Thus, regardless of the level, every hierarchical cell on Earth has a unique global ID assigned. In other words, Google Plus Code established a reference system with unique ID for every cell in a hierarchical grid system. Google (google.com/maps or plus.codes/map) can provide location and direction to any specific cell directly with its ID. For example, when searching “8P4W3VQC+”, Google Map will show the central point of the cell and plus.codes will show the cell boundary on the map. Along a similar latitude, the cells of this well-defined grid system have same area and provide foresters a useful spatial unit to locate, analyze and compare forest resources at the global level. Cell size does vary at different latitudes due to different ground distances corresponding to same unit of longitude. For example, the latitude ranges from 32.041° to 32.091° for our study area, cells area change from 65,455 m^2^ on the south side to 65,430 m^2^ on the north side. The minor difference, 25 m^2^, is ignorable. A coefficient can be used if cells at significant different latitudes need to be compared. It is worth mentioning that the open source nature of Plus Code enables it to be directly used within any Geographic Information Systems (GIS), such as ArcGIS^®^ and QGIS. Level 3 Plus Code grid (8 digits ID) is used for the study area in this research in order to compare the spatial change of forest carbon storage.

The forest carbon storage CV index (Coefficient of Variation) can be used to represent the relative stability of a cell’s forest carbon storage. The larger the CV index, the less stable the cell’s forest carbon storage is. On the contrary, the smaller CV index means a more stable forest carbon storage. CV index can be calculated with the equation below:

$CV=\frac{\sqrt{\frac{1}{n}\sum_{i=1}^{n} \left( C_{i}-\overline{C} \right)^{2}}}{\overline{C}}$ （4）

C_i_ is the forest carbon storage at year i and is the average forest carbon storage for each cell from 1987 to 2019.

Level 2, 3, and 4 grid cells have an area of 2630, 6.6 and 0.016 ha, respectively. For carbon storage and forest management, level 3 cells of Plus Code were used as the location and unit boundary for this study. There are 588 cells and each cell has an area of 66,123 m^2^ (237 m × 279 m). At this level, the ID for each cell has 8 digits, such as 8P4W3VQC (Figure 2). For every forest inventory survey year, the carbon storage of each cell was estimated with ArcGIS 10.5^®^ based on the methodology described in this section. Proportional method was applied for cells with multiple sub-compartments.

**Results**

Forest carbon storage change

Forest area in the park (including all tree species and bamboo forest) increased from 1791.5 ha in 1987 to 2,311.1 ha in 2019 (Figure 3). This increase in forest area is noteworthy particularly given the increasing number of visitors per year to the park and the rapid urbanization and population growth in the Nanjing metropolitan area over that time period. The rate of increase in forest area from 2002 to 2019 (207 ha) is lower than that from 1987 to 2002 (312 ha) due to the higher initial reforestation efforts that occurred in the park in the late 20th century. Mixed conifer and broadleaf forest area increases accounted for most of this change. Forest carbon storage also increased for the study area (Figure 2). However, the rate of forest carbon storage from 2002 to 2019 is higher than that from 1987 to 2002 due to the forest type and age structure. For the 32-year study period, the net increase in forest carbon storage was 43,740.7 tC. The average annual increase in forest carbon storage was 1,197.6 tC/year and 1,365.5 tC/year for the period of 1987 to 2002 and 2002 to 2019, respectively.


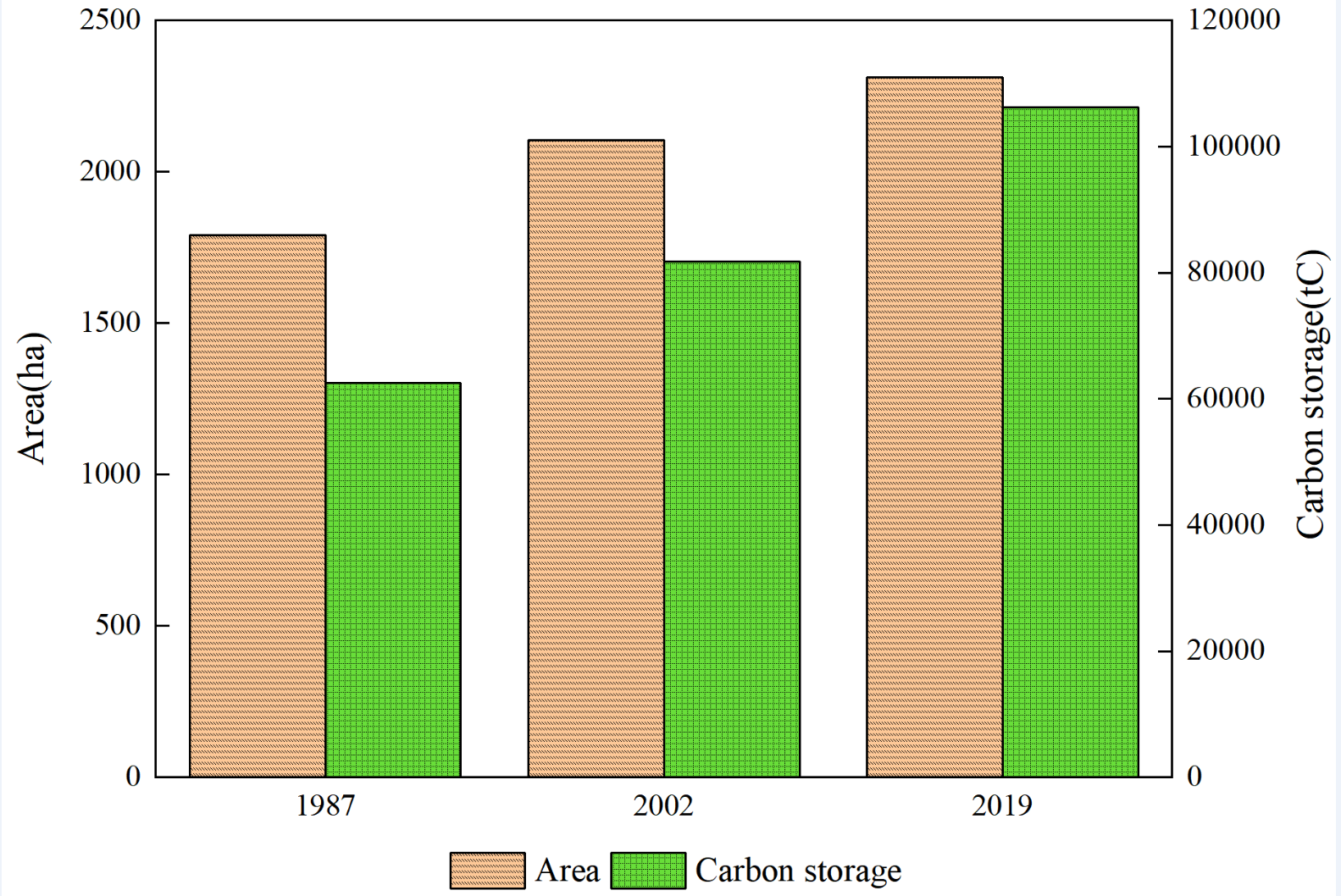


**Fig. 3** Change of forest area and forest carbon storage in the Zijin Mountain National Forest Park from 1987 to 2019

Changes in forest carbon corresponded to different forest types and varied among measurement periods (Table 2). For example, broadleaf forests accounted for just over half (51-54%) of the carbon stored per ha compared to conifer which accounted for approximately a quarter (24-28%). Bamboo remained relatively constant throughout the study period both in terms of area (2%) and the percentage of the carbon stored per ha (11-14%). The mixed conifer/broadleaf forest type increased the most in terms of forest area, from 8% (126 ha) of the park in 1987 to 31% (443 ha) by 2019. This mostly occurred as stands of pure Masson pine forests were converted into mixed forest types, with Masson pine area decreasing from 444 ha in 1987 to 32 ha in 2019 due to insect mortality. However, the mixed conifer/broadleaf forest type accounted for the lowest proportion of carbon storage, ranging from only 8-9% of the tons of carbon per ha from 1987-2019. This lower percentage of the per ha carbon storage can in part be explained by the younger age class of this forest cohort, as noted below.

Table 2 Carbon storage and carbon density of different forest types in the Zijin Mountain National Forest Park from 1987 to 2019

| year | 1987 | | | | 2002 | | | | 2019 | | | |
| --- | --- | --- | --- | --- | --- | --- | --- | --- | --- | --- | --- | --- |
| type | area  (ha^)^ | Carbon storage | | cd^*^  (tC/ha) | Area  (ha) | Carbon storage | | cd  (tC/ha) | Area  (ha) | Carbon storage | | cd  (tC/ha) |
|  |  | tC | % |  |  | tC | % |  |  | tC | % |  |
| Oak (*Quercus*) | 368.8 | 16,285.4 | 26.0 | 44.2 | 553.8 | 26,947.1 | 33.0 | 48.7 | 518.3 | 31,042.0 | 29.2 | 59.9 |
| Formosan gum  （*Liquidambar formosana*） | 102.8 | 3,924.3 | 6.3 | 38.2 | 177.4 | 6,957.6 | 8.5 | 39.2 | 215.9 | 7,895.5 | 7.4 | 36.6 |
| Locust  (*Robinia pseudoacacia*) | 155.8 | 5,803.9 | 9.3 | 37.3 | 74.7 | 2,910.3 | 3.6 | 39.0 | 49.4 | 2,266.0 | 2.1 | 45.9 |
| Masson pine  (*Pinus massoniana Lamb.*) | 444.4 | 13,180.7 | 21.1 | 29.7 | 215.3 | 8,379.7 | 10.3 | 38.9 | 32.3 | 1,404.0 | 1.3 | 43.4 |
| Mixed broadleaves | 395.0 | 12,838.3 | 20.5 | 32.5 | 635.8 | 22,792.4 | 27.9 | 35.8 | 968.9 | 45,601.3 | 42.9 | 47.1 |
| Mixed  (Conifers and broadleaves) | 126.4 | 3,002.4 | 4.8 | 23.8 | 367.9 | 10,039.0 | 12.3 | 27.3 | 433.4 | 13,507.2 | 12.7 | 31.2 |
| Mixed conifers | 173.8 | 6,500.4 | 10.4 | 37.4 | 48.0 | 2,419.7 | 3.0 | 50.4 | 56.2 | 3,065.8 | 2.9 | 54.5 |
| Bamboo  (*Phyllostachys pubescens*) | 24.6 | 1,003.3 | 1.6 | 41.0 | 30.7 | 1,257.2 | 1.5 | 41.0 | 36.6 | 1,498.8 | 1.4 | 41.0 |
| Total | 1,791.5 | 62,538.7 | 100.0 |  | 2,103.6 | 81,703.0 | 100.0 |  | 2,311.0 | 106,280.6 | 100.0 |  |

*cd stands for carbon density

Across all forest types, the mixed conifer class had the greatest increase in carbon storage per ha, increasing by 17.1 tC/ha from 37.4 tC/ha in 1987 to 54.5 tC/ha in 2019. For the broadleaves forest, oak had the greatest increase in rate of carbon storage, steadily increasing by 15.7 tC/ha from 44.2 tC/ha in 1987 to 59.9 tC/ha 2019. The rate of carbon storage for Masson pine increased by 13.7 tC/ha and 8.6 tC/ha locust from 1987 to 2019. Formosan gum remained fairly consistent in its rate of carbon storage, decreasing slightly by 1.6 tC/ha. Carbon storage rates are related to stand age structure in addition to species, as noted below. Bamboo was the most consistent cover type in the park in terms of carbon storage per ha in part because of its more rapid maturation rate when compared with tree species.

Forest Carbon Storage by Age Cohort

As noted above, forest carbon storage is dependent on forest age structure. Age classification is based on tree species and cover type which were derived from province level forest management practices (Table 3).

Table 3. Forest age classification (years) for forest cover types in the Zijin Mountain National Forest Park in Jiangsu Province, China.

| Forest type | Young forest | Middle aged forest | Near mature forest | Mature forest | Over matured forest |
| --- | --- | --- | --- | --- | --- |
| Locust | ≤10 | 11-20 | 21-30 | 31-50 | >50 |
| Oak | ≤20 | 21-40 | 41-50 | 51-70 | >70 |
| Formosan gum | ≤10 | 11-20 | 21-30 | 31-50 | >50 |
| Masson pine | ≤10 | 11-20 | 21-30 | 31-50 | >50 |
| Mixed conifers | ≤10 | 11-20 | 21-30 | 31-50 | >50 |
| Mixed broadleaves | ≤20 | 21-40 | 41-50 | 51-70 | >70 |
| Mixed (mixed Conifers and broadleaves) | ≤10 | 11-20 | 21-30 | 31-50 | >50 |

Forests in this park are primarily managed for their scenic value and recreation opportunities for visitors and there was no forest harvesting in the study area during this 32-year study period. Overall, stand cohorts aged by 32 years and there was an estimated net increase in total accumulated carbon in the park with this increase in biomass (Table 4 and Figure 4). For example, young forest carbon density increased from 17.7 tC/ha to 22.5 tC/ha and 31.0 tC/ha for 1987, 2002, and 2019, respectively. This can be attributed to hardwood reforestation in the study area over the years. Similarly, in 1987, middle-aged and mature forests covered 76.7% of the area and correspondingly, their carbon storage accounted for about the same proportion: 76.4% of the total storage. In 1987, over-mature forest occupied only a small area with little carbon storage (0.2%). By 2002 and 2019, the over-mature forest area increased as did the total carbon stored in these forests, accounting for 16.5% (2002) and 30.0% (2019) of total storage. These results are consistent with those reported by Chen et al. [18] for southeastern China. For the current study, the rate of accumulation by forest type for over-mature forests was not evaluated due to data limitations. That said, consistent with the findings of Chen et al. [18], as over-mature forests begin to senesce in growth rate, the rate of carbon accumulation per ha will likely continue to decrease. This may necessitate some forest harvesting and management to prevent these over-mature forests from releasing this stored carbon as they decay in the coming decades [19].

Table 4. Carbon storage and carbon density of different age groups in the Zijin Mountain National Forest Park from 1987 to 2019

| year | 1987 | | | | 2002 | | | | 2019 | | | |
| --- | --- | --- | --- | --- | --- | --- | --- | --- | --- | --- | --- | --- |
| Age groups | Area  ha | Carbon storage | | Carbon density  tC/ha | Area  ha | Carbon storage | | Carbon density  tC/ha | Area  ha | Carbon storage | | Carbon density tC/ha |
|  |  | tC | % |  |  | tC | % |  |  | tC | % |  |
| Young | 98.4 | 1,744.8 | 2.8 | 17.7 | 197.1 | 4,439.6 | 5.5 | 22.5 | 51.1 | 1582.6 | 1.5 | 31.0 |
| Middle-aged | 675.9 | 25,807.3 | 41.9 | 38.2 | 557.6 | 22,081.1 | 27.4 | 39.6 | 479.0 | 21,205.1 | 20.2 | 44.3 |
| Near-mature | 311.9 | 12,666.9 | 20.6 | 40.6 | 353.1 | 14,080.4 | 17.5 | 39.9 | 360.2 | 16411.7 | 15.7 | 45.6 |
| Mature | 678.7 | 21,207.4 | 34.5 | 31.3 | 644.9 | 26,541.1 | 33.0 | 41.2 | 709.9 | 34118.6 | 32.6 | 48.1 |
| Over-mature | 2.1 | 109.0 | 0.2 | 51.1 | 320.2 | 13,303.7 | 16.5 | 41.5 | 674.2 | 31463.8 | 30.0 | 46.7 |
| total | 1,767.0 | 61,535.4 | 100.0 | 34.8 | 2,072.9 | 80,445.9 | 100.0 | 38.8 | 2,274.4 | 104,781.8 | 100.0 | 46.1 |


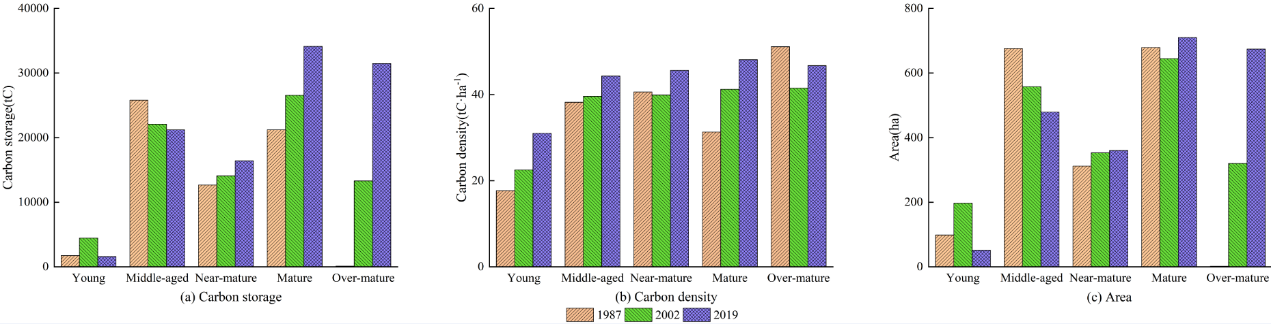


**Fig. 4** Dynamic changes of area, carbon storage and carbon density of each age group in the Zijin Mountain National Forest Park from 1987 to 2019

Analysis of forest carbon storage distribution based on Plus Code cells

As mentioned before, 588 Plus Code level 3 grid cells (237 m × 279 m) were used as the location and unit boundary for the study area. For every forest inventory survey year, the carbon storage of each cell was calculated and classified into 8 categories (Figure 5). The entire area was then grouped into low storage area (less than 200 tC/cell), medium storage area (200 – 400 tC/cell) and high storage area (400 – 800 tC/cell) (Table 5). Forest carbon storage is greater for the northern cells which is more heavily forested since there is a greater proportion of scenic sites and tourist areas in the southern part. From a temporal aspect, carbon storage continued increasing, especially for the southern cells during the past 32 years. The coverage of medium storage area increased from 44.8% (1987) to 49.4% (2002) and 52.0% (2019). The coverage of high storage area increased from 0.3% (1987) to 4.5% (2002) and 8.3% (2019). In summary, the total forest carbon storage increased from 1987 to 2019, as would be expected given the forest restoration efforts and improved management practices in the park.


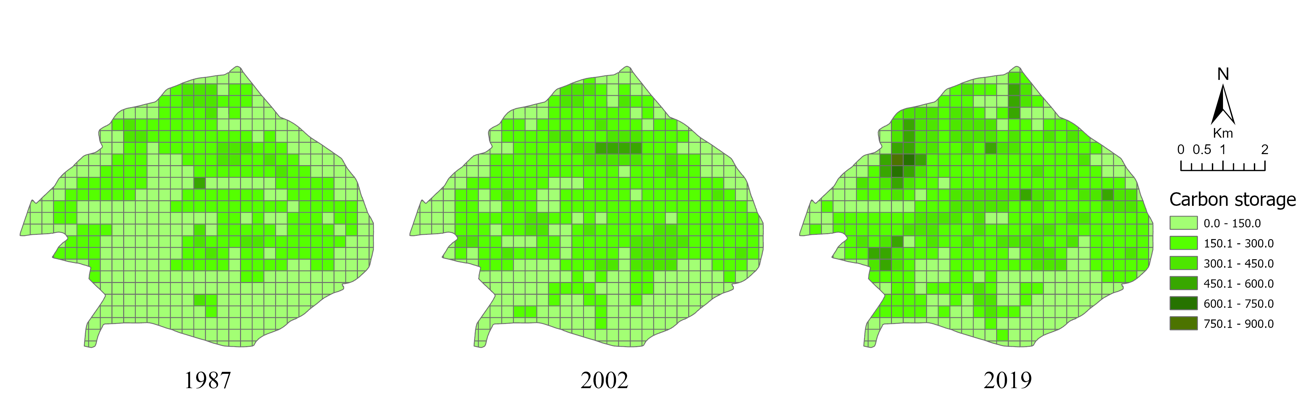


**Fig. 5** Forest carbon storage by Plus Code level 3 cell in the Zijin Mountain National Forest Park from 1987 to 2019

Table 5. Areas of forest carbon storage at different levels in the Zijin Mountain National Forest Park from 1987 to 2019

| Carbon storage  (tC) | level | 1987 | | 2002 | | 2019 | |
| --- | --- | --- | --- | --- | --- | --- | --- |
|  |  | Area  (ha) | Percentage  (%) | Area  (ha) | Percentage  (%) | Area  (ha) | Percentage  (%) |
| [0,200) | Low | 983.3 | 54.9 | 970.1 | 46.1 | 918.4 | 39.7 |
| [200,400) | Medium | 803.2 | 44.8 | 1038.3 | 49.4 | 1200.6 | 52.0 |
| [400,800) | High | 5.1 | 0.3 | 95.2 | 4.5 | 192.1 | 8.3 |

Plus Code cells were classified into 5 categories based on carbon storage CV index, with low stability (13.3%), low-medium stability (12.8%), medium stability (12.9%), medium-high stability (33.1%), and high stability (25.1%) (Table 6 and Figure 6). In terms of stability, 71.1% of the forest is in the category of medium stability or greater, indicating that the forest carbon storage of the entire park is relatively stable. Most of the cells with low stability cells are on the south facing slopes, where forest carbon storage increased most from 1987 to 2019. Cells with relatively small CV Index tend to be located on the north facing slope where rates of change have been lower. Thus, forest carbon storage changed less and is relatively stable.

Table 6. CV Index classification of forest carbon storage in the Zijin Mountain National Forest Park from 1987 to 2019

| CV Index | Stability | Area (ha) | Coverage (%) |
| --- | --- | --- | --- |
| [0.8,1.4] | low | 400.5 | 13.3 |
| [0.6,0.8) | low-medium | 384.8 | 12.8 |
| [0.4,0.6) | medium | 388.0 | 12.9 |
| [0.2,0.4) | medium-high | 993.6 | 33.1 |
| [0,0.2) | high | 753.4 | 25.1 |


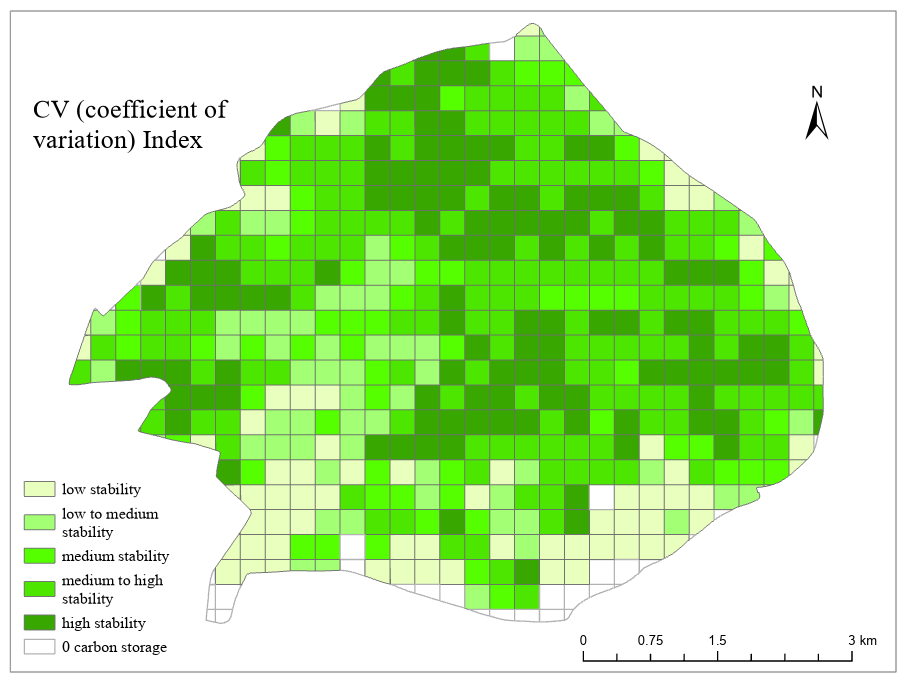


**Fig. 6** CV Index distribution of forest carbon storage in the Zijin Mountain National Forest Park from 1987 to 2019

**Discussion**

As pointed out by Nabuurs et al. [20], to mitigate climate change, there are sill knowledge gaps in the monitoring and modeling of restoration efforts. We need to understand the interaction of forest management and carbon storage better through historical data and new technology. This research tried to closing the knowledge gap by quantification of forest carbon storage with the new Google Plus Code spatial grid unit. The temporal and spatial dynamics of forest carbon storage and density were evaluated in the Zijin Mountain National Forest Park based on the three forest inventories in 1987, 2002 and 2019. A newly available alphanumeric geocoding method, Google Plus Code, was used to define spatial units and these forest inventories were analyzed to estimate carbon storage across the park by location. Conclusions drawn from this analysis include first, from a temporal aspect, forest coverage, carbon storage and carbon density all increased from 1987 to 2019. Carbon storage increased by 43,740.7 tC and the average annual increase was 1,325.5 tC. Overall, Zijin Mountain National Forest Park became a more stable carbon sink. From 1987 to 2019, carbon storage in broadleaf forests increased the most. Coniferous forest decreased, especially Masson pines, mainly due to the heavy mortality of pines from insects and the subsequent regeneration of broadleaf species such as oaks and Formosan gum. By age cohort, middle-aged and mature forests accounted for most (more than 60%) of the carbon storage in 1987 and 2002. Forests continued to age and mature as a result of forest restoration and protection efforts. Thus, middle-aged forests became mature and/or over-mature and carbon storage reached 59.5% of the total amount. In the past 32 years, the carbon density of young, middle-aged and near-mature forests increased constantly while the carbon density of matured and over matured forests declined. Additional forest inventories should be conducted to determine the age at which over-mature forests transition from carbon sinks to potential carbon sources. Forest conservation and management management measures should be used to maintain and enhance carbon storage along with the other ecosystem services that the forests provide in this park.

In addition, from the geospatial aspect, Google Plus Code was found to be an effective grid system for analyzing temporal and spatial trends in carbon storage. Using this system, it was found that the southern portion of the study area had less carbon storage than the northern portion in 1987 and 2002. However, carbon storage increased by 2019. Medium and high carbon storage area coverage increased from 45.1% (1987) to 53.9% (2002) and 60.3% in 2019. The Google Plus Code analysis indicated that perhaps future restoration and forest protection efforts should be focused in this area. Pairing Google Plus Code with the CV index showed that the carbon storage stability is relatively high for entire study area.

One last thing worth mentioning is that Google Plus Code is a system in the new filed of Discrete Global Grid System (DGGS) which is supported by the Open Geospatial Consortium (OGC) and the International Organization for Standardization (ISO) [21]. We hope this case study can lead to more applications of global grid system for spatial-temporal information, such as carbon distribution and storage.

**Conclusions**

In conclusion, foresters are increasingly needing to manage forests for enhanced carbon sequestration potential as this ecosystem service increases in both economic and environmental value. From the forest management and monitoring aspect, this case study illustrates how foresters and land managers can overcome a persistent problem that results from the boundaries of compartments and sub-compartments being modified by changing forest conditions, from natural disasters to forest fragmentation that occurs with land ownership changes. Google Plus Code grid has geospatial location/area information embedded with a unique global ID to which forest conditions can be locked to a particular spatial unit in time. This enables more efficient quantification of changes in land use and forest conditions. This case study was focused an important concern for China, carbon storage and sequestration through forest restoration in one of its iconic natural areas, Zijin Mountain National Forest Park. Google Plus Code can be a valuable tool to help foresters to understand and monitor the impact of forest management activities on carbon storage with high-resolution temporal and spatial accuracy. This methodolgy can also provide technical support for carbon credit management. Additional studies are needed to assess the potential of Google Plus Code for the other aspects of ecosystem management, including but not limited to the biomass of litter, understory, grass, below-ground biomass, and water resources to provide a more comprehensive picture of the eco-function that forests provide. Future research can include but not limited to uncertainty analysis, applying this method to compare carbon storage at different locations at different scale, and studying the impact of forest management practices on carbon storage.

**Declarations**

**Ethics approval and consent to participate**

Not applicable.

**Consent for publication**

Not applicable.

**Availability of data and materials**

Forest inventory data are available upon request.

**Competing interests**

The authors declare that they have no competing interests.

**Funding**

The research was funded by Forestry Innovation Foundation of Guangdong Province (No.2021KJCX001), Priority Academic Program Development of Jiangsu Higher Education Institutions (PAPD) and the National Key R&D Program of China (2016YFC0502704).

**Authors' contributions**

XW and YZ developed the method and validated the results. XW, YL, YZ, QW, and JY worked on data collection and analysis. XW, YZ, and MM wrote and edited the manuscript. All authors reviewed the manuscript.

**Acknowledgements**

The authors thank the Zijin Mountain National Forest Park for allowing forest data collection throughout the years. Also thanks to an anonimous reviewer who provided very constructive suggestions and comments.

**References**

1. Yang Y, Shi Y, Sun W, Chang J, Zhu J, Chen L, et al. Terrestrial carbon sinks in China and around the world and their contribution to carbon neutrality. Sci China Life Sci. 2022;65(5):861–95. http://dx.doi.org/10.1007/s11427-021-2045-5
2. McKinley DC, Ryan MG, Birdsey RA, Giardina CP, Harmon ME, Heath LS, et al. A synthesis of current knowledge on forests and carbon storage in the United States. Ecol Appl . 2011;21(6):1902–24. http://dx.doi.org/10.1890/10-0697.1
3. Woodbury PB, Smith JE, Heath LS. Carbon sequestration in the U.S. forest sector from 1990 to 2010. Forest Ecological Management. 2007;241(1–3):14–27. http://dx.doi.org/10.1016/j.foreco.2006.12.008
4. Fang J, Chen A, Dynamic forest biomass carbon pools in China and their significance. J. of Integrative Plant Biology. 2001 Jan 1;43(9):967.
5. Fang J, Chen A, Peng C, Zhao S, Ci L. Changes in forest biomass carbon storage in China between 1949 and 1998. Science. 2001;292(5525):2320–2. http://dx.doi.org/10.1126/science.1058629
6. Yang J, Ji X, Deane D, Wu L, Chen S. Spatiotemporal distribution and driving factors of forest biomass carbon storage in China: 1977–2013. Forests. 2017;8(7):263. http://dx.doi.org/10.3390/f8070263
7. He C, Wang S, Xu J, Zhou C. Using remote sensing to estimate the change of carbon storage: A case study in the estuary of Yellow River delta. Int J Remote Sens. 2002;23(8):1565–80. http://dx.doi.org/10.1080/014311602753590887
8. Sun YS, Wang WF, Li GC. Spatial distribution of forest carbon storage in Maoershan region, Northeast China based on geographically weighted regression kriging model. J. of Applied Ecology. 2019;30(5):1642–50. http://doi.org/10.13287/j.1001-9332.201905.024
9. Zhang Y, Stovall J, Unger D, Hung I, Kulhavy D. Plus Code: an Alphanumeric code geocoding option for professional foresters, The Forestry Source. 2022;27(2):1
10. Jandl R, Vesterdal L, Olsson M, Bens O, Badeck F, Roc J. Carbon sequestration and forest management. CABI Reviews. 2007 Jun 20(2007):16-pp.
11. Pan Y, Birdsey RA, Fang J, Houghton R, Kauppi PE, Kurz WA, et al. A large and persistent carbon sink in the world’s forests. Science. 2011;333(6045):988–93. http://dx.doi.org/10.1126/science.1201609
12. Liu Q, Ouyang Z, Li A, Xu W. Spatial Distribution Characteristics of Biomass and Carbon Storage in Forest Vegetation in Chongqing Based on RS and GIS. Nature Environment & Pollution Technology. 2016 Dec 1;15(4).
13. Zhang C, Ju W, Chen JM, Zan M, Li D, Zhou Y, et al. China’s forest biomass carbon sink based on seven inventories from 1973 to 2008. Climate Change. 2013;118(3–4):933–48. http://dx.doi.org/10.1007/s10584-012-0666-3
14. Zhao M, Zhou GS. Estimation of biomass and net primary productivity of major planted forests in China based on forest inventory data. Forest Ecological Management. 2005;207(3):295–313. http://dx.doi.org/10.1016/j.foreco.2004.10.049
15. Liu G, Fu B, Fang J. Carbon dynamics of Chinese forests and its contributions to global carbon balance. Acta Ecologica Sinica, 2000, Jan 1;20(5):733-740.
16. China National Forestry and Grassland Administration (CNFGA), 2014. Guide for Carbon Accounting and Monitoring in Afforestation Projects, LY/T 2253-2014.
17. Guo Z, Hu H, Li P. Spatial-temporal changes in biomass carbon sinks in China's forests from 1977 to 2008. Science China Life Sciences, 2013;56(7):661-671 http://dx.doi.org/10.1007/s11427-013-4492-2
18. Chen LC, Liang MJ, Wang SL. Carbon stock density in planted versus natural Pinus massoniana forests in sub-tropical China. Ann For Sci . 2016;73(2):461–72. http://dx.doi.org/10.1007/s13595-016-0539-4
19. Tang J, Bolstad PV, Desai AR, Martin JG, Cook BD, Davis KJ, et al. Ecosystem respiration and its components in an old-growth forest in the Great Lakes region of the United States. Agricultural and Forest Meteorology. 2008;148(2):171–85. <http://dx.doi.org/10.1016/j.agrformet.2007.08.008>
20. Nabuurs GJ, Mrabet R, Hatab AA, Bustamante M, Clark H, Havlík P, House JI, Mbow C, Ninan KN, Popp A and Roe S. Agriculture, forestry and other land uses (AFOLU). In Climate Change 2022: Mitigation of Climate Change. (pp. 747-860). 2022. Cambridge University Press.
21. Gibb R. Purss M. Sabeur Z. Strobl P. Qu T. Global reference grids for big earth data. Big Earth Data. 2022;6(3):251-5
